# Differential transferrin expression in placentae from normal and abnormal pregnancies: a pilot study

**DOI:** 10.1186/1477-7827-6-27

**Published:** 2008-07-02

**Authors:** Alena Kralova, Marta Svetlikova, Jindrich Madar, Zdena Ulcova-Gallova, Antonin Bukovsky, Jana Peknicova

**Affiliations:** 1 Institute of Biotechnology, Academy of Sciences of the Czech Republic, v.v.i., 142 20, Prague, Czech Republic; 2 The University of Tennessee Graduate School of Medicine, Knoxville, TN 37920, USA; 3 Institute for the Care of Mother and Child, 147 10, Prague, Czech Republic; 4 Department of Gynaecology and Obstetrics, 304 60, Pilsen, Czech Republic

## Abstract

**Background:**

The placenta is an important site for iron metabolism in humans. It transfers iron from the mother to the fetus. One of the major iron transport proteins is transferrin, which is a blood plasma protein crucial for iron uptake. Its localization and expression may be one of the markers to distinguish placental dysfunction.

**Methods:**

In the experimental study we used antibody preparation, mass spectrometric analysis, biochemical and immunocytochemical methods for characterization of transferrin expression on the human choriocarcinoma cell line JAR (JAR cells), placental lysates, and cryostat sections. Newly designed monoclonal antibody TRO-tf-01 to human transferrin was applied on human placentae from normal (n = 3) and abnormal (n = 9) pregnancies.

**Results:**

Variations of transferrin expression were detected in villous syncytiotrophoblast, which is in direct contact with maternal blood. In placentae from normal pregnancies, the expression of transferrin in the syncytium was significantly lower (p < 0.001) when compared to placentae from abnormal ones (gestational diabetes, pregnancy induced hypertension, drug abuse).

**Conclusion:**

These observations suggest that in the case of abnormal pregnancies, the fetus may require higher levels of transferrin in order to prevent iron depletion due to the stress from the placental dysfunction.

## Background

All cells and organisms require iron to perform basic cellular processes. Its importance in respiration and oxygen transport led to the evolution of an effective transport system of iron ions throughout the organism. This transport is mediated by transferrin (TF), serum protein produced especially by hepatocytes. Transferrin (TF; PRO2086) is a glycoprotein with a molecular mass of 76–80 kDa carrying homologous C- and N- terminal iron-binding domains [1]. Bound ferric iron ions are transported from the intestine, reticuloendothelial system, and liver parenchymal cells through the blood to all proliferating cells in the body. The process is based on receptor-mediated endocytosis. After nonlysosomal (endosomal) dissociation of iron, transferrin and its receptor return to the extracellular environment and the cell membrane, respectively [2].

Aside from their essential synthesis in the liver, transferrins are also described to be produced locally in the testes (Sertoli cells) and the central nervous system (brain glial-cells, choroid plexus) [3], as well as in fetal membranes and the placenta [4], which are areas relatively inaccessible to proteins in the general circulation. As all cells require iron from serum transferrin produced by hepatocytes, cells that create the blood barrier of the testes, brain and placenta tissue express the transferrin gene to provide iron to cells sequestered within the serum-free environment [5]. As for embryo development, the growing fetus needs increasing amounts of iron, which is provided by its release from maternal transferrin. It is responsible for the transport of iron to cells within both the fetal and maternal systems, but it does not cross the multiple cell layer barrier of the placenta. Recent findings that human placental cells produce TF indicated that placental TF may be involved in the transport or regulation of iron passage across this barrier [6]. Iron deficiency anemia early in pregnancy doubles the risk of preterm delivery [7], while fetal anemia may contribute to the development of cardiovascular disease in adulthood [8]. Understanding how iron is transported through the placenta is important in this context. However, placental production of transferrin still remains to be clarified [6].

In our laboratory, we prepared antibodies targeted against selected placental proteins. The monoclonal antibody designated TRO-tf-01 was shown to specifically recognize one isoform of transferrin in the human placenta. This paper describes the expression differences of this isoform of transferrin in placental samples with respect to their diverse physiological statuses. The evaluation of changes in transferrin expression could help to understand the placental adaptive mechanisms involved in compensating the function in abnormal pregnancies.

## Methods

### Sample collecting

Term placentae of three normal (NP) and nine abnormal pregnancies of mothers admitted to the University of Tennessee Medical Center to deliver were examined by indirect peroxidase immunohistochemistry. Excluded were patients with blood transferable infections, e.g., hepatitis and HIV, and apparent ascendant placental infections. The study was approved by the Institutional Review Board and all patients signed the "Informed Consent". For the study, some abnormalities of placentae were selected. A group of abnormal pregnancies consisted of gestational diabetes (GD, n = 3), pregnancy-induced hypertension (PIH, n = 3), and drug abuse (crack, cocaine) during pregnancy (DrA, n = 3).

### Sample preparation, extraction

For biochemical analysis, samples of placental tissue were treated as described elsewhere [9]. Human choriocarcinoma cell line (JAR cells) was cultivated in RPMI medium (RPMI 1640, Sevapharma, Prague, Czech Republic) supplemented with 10% fetal bovine serum (FBS, Sigma, Prague, Czech Republic) for three days. After their removal with ethylenediaminetetraacetic acid (EDTA), the cells were washed three times (centrifugation at 300 × g for 15 min at 25°C) in phosphate-buffered saline (PBS, 20 mM phosphate buffer, 0.15 M NaCl, pH 7.4). Suspension of washed cells was centrifuged at 1000 × g (10 min at 4°C) and the pellet was resuspended in sodium dodecyl sulfate (SDS) (twice concentrated non-reducing SDS sample buffer) or by adding an ice-cold lysis buffer (20 mM Tris pH 7.5, 0.20 M NaCl, 0.25% Nonidet P-40; 400 μl/100 mg of cell mass or placental tissue) containing 1 mM of sodium orthovanadate, 10 mM of sodium fluoride, and 1 mM of phenylmethylsulfonyl fluoride [9]. After heating in a boiling water bath for 3 min (SDS extraction) or after 15 min on ice (Nonidet P-40 extraction), the lysates were sonicated using a SonicatorTM Cell Disruptor (Heat Systems-Ultrasonic, Inc., Plainview, NY) for 5 seconds, and centrifuged (at 15000 × g for 20 min at 4°C), and the aliquots of lysates were deep-frozen at -70°C. For Western blot analysis, protein concentrations were determined by BCA Bradford assay (Bio-Rad, Hercules, CA).

### Immunization and monoclonal antibody preparation

Monoclonal antibody TRO-tf-01 was prepared by the immunization of BALB/c mice (Velaz, Prague, Czech Republic) with JAR cells. Mice were twice subcutaneously immunized within 10 days with 50 μg of total protein in 75 μl 1:1 crude extract: complete Freund adjuvant (CFA, Sigma, Prague, Czech Republic). A booster injection of 150 μl 1:1 crude CFA extract was applied intraperitoneally 10 days after the second immunization. The volume was equivalent to 100 μg of total protein. Three days after the third immunization, hyperimmune spleen cells were fused with myeloma cells Sp2/0. Positive clones were selected by enzyme-linked immunosorbent assay (ELISA). Standard protocols for hybridoma production, selection, cloning and specificity controls were performed as described before [10].

### Monoclonal antibody isotyping kit

For the isotype characterization of the TRO-tf-01 monoclonal antibody, a mouse monoclonal antibody isotyping kit (IsoStrip, Roche, Indianapolis, USA) was used according to the manufacturers instructions.

Briefly, a supernatant of TRO-tf-01 antibody was diluted in PBS (1:50). A freshly diluted sample (150 μl) was pipetted into a development tube, and agitated so that the colored latex beads were completely resuspended. An isotyping strip was placed in the tube. Within 5 min, a blue band appeared, indicating the class or subclass and light-chain composition of the monoclonal antibody.

### Enzyme-linked immunosorbent assay (ELISA)

One hundred μl of individual JAR extracts diluted by phosphate-buffered saline (PBS, 20 mM phosphate buffer, 0.15 M NaCl, pH 7.4) to a concentration of 0.05 – 1.0 μg/μl were used for coating the wells of a 96-well microplate (overnight at 4°C). After coating, the plate was washed three times with PBST (PBS, 0.05% Tween 20), and 100 μl bovine serum albumin – phosphate-buffered saline-Tween (BSA-PBST) (PBS, 1% BSA, 0.05% Tween 20) were added for 1 hour at 25°C to block nonspecific binding sites. After triple washing with PBST, 100 μl of MoAb TRO-tf-01 supernatant, serially diluted in BSA-PBST, were added to the appropriate wells and the plate was incubated for 1 hour at 37°C. After the antibody-antigen reaction, the plate was washed three times with PBST, and 100 μl (1.4 μg) of peroxidase-conjugated swine anti-mouse antibody (SWAM/Px, Sevapharma, Prague, Czech Republic) were added to each well. The plate was then incubated for 1 hour at 37°C. The reaction was stopped by adding 4 N H_2_SO_4_. The peroxidase activity was detected with a Sunrise Absorbance Reader (Tecan Trading AG, Switzerland) at 492 nm using H_2_O_2 _and o-phenylenediamine (Fluka, Buchs, Switzerland) after triple washing with PBST.

### SDS-PAGE and immunoblotting

Sodium dodecyl sulfate polyacrylamide gel electrophoresis (SDS-PAGE) was performed according to the method of Laemmli [11]. Equal amounts (20 μg) of boiled protein (3 min at 100°C) were loaded onto reducing 10% SDS-Tris-glycine polyacrylamide gels. Electrophoresis proceeded in electrode buffer (0.25 M Tris, 1.92 M glycine, 10% SDS) at a constant amperage of 16 mA/gel. The molecular mass of proteins banded in the gels was estimated using molecular weight markers (Sigma, Prague, Czech Republic) run parallelly.

For immunodetection, the separated proteins were then electrophoretically transferred onto Hybond C-super nitrocellulose membranes (Amersham Biosciences, Uppsala, Sweden) according to the arrangement described by Towbin *et al*. [12]. Electrophoretical transfer was carried out in a Tris-glycine transfer buffer (pH 9.6) with 20% (v/v) methanol in an LKB Transpher System at a starting amperage of 0.5 mA/cm^2 ^(2 h at 4°C).

In 2-D electrophoresis experiments, isoelectric focusing was performed according to the standard manufacturer's protocol (Immobiline DryStrip Instruction manual, GE Healthcare, Prague, Czech Republic). The total protein lysate of the placental tissue (180 μg) was applied during rehydratation of the 13 cm pH 4–7 Immobiline DryStrip (Ettan IPGphor 3, GE Healthcare, Prague, Czech Republic). In the second dimension, 10% polycrylamide slab gel (SDS) was used.

In immunodetection, the blocking of the membranes was done overnight with 5% gelatin (Sigma, Prague, Czech Republic) in PBST (PBS, 0.05% Tween 20) at 4°C. The membranes were incubated with a supernatant of MoAb TRO-tf-01 (1:200 dilution in 1% gelatin-PBST) at 25°C for 1 hour. After five 5-minute washes in PBST, the membranes were incubated with horseradish peroxidase (HRP)-conjugated goat anti-mouse antibody (GAM/Px) (Bio-Rad, Prague, Czech Republic) diluted 1:3000 in 1% gelatin-PBST for 1 hour at 25°C. The membrane was washed again in PBST, and chemiluminescent substrate (SuperSignal, Pierce, Rockford, USA) was applied for visualization of the corresponding bands.

To ascertain equal protein loading, the blot was reprobed with mouse monoclonal anti-actin antibody (pan, Ab-5; NeoMarkers, Fremont, CA, 0.5 μg/ml) and developed with goat anti-mouse IgG and IgM (Jackson Immunoresearch, West Grove, PA) diluted 1:5000 for 1 hour at 25°C.

Band densities were quantified by densitometry using the Fluorchem digital imaging system (Alpha Innotech Co., San Leandro, CA). The resulting crude optical density (O.D.) was subtracted from the mean of O.D. of the background.

### CBB staining

Gels destined to mass spectrometric analysis were stained with Coomassie Brilliant Blue (CBB) R-250 (Serva, Germany) for vizualization of all separated proteins. After SDS-PAGE, the gels were incubated at room temperature in a solution containing CBB (0.25% CBB R-250, 7% CH_3_COOH, 50% ethanol) for 1 hour. After incubation with CBB, the gels were washed in destaining solution (35% ethanol, 10% CH_3_COOH) until the background dissapeared and the separated proteins were clearly and sharply visible.

### Mass spectrometric analysis

The spots of interest were excised from the Coomassie R-250- stained SDS-PAGE gel. Destaining, digestion with trypsin, and preparation of the resulting protein mixtures for mass spectrometric analysis were performed in accordance with Kovarova *et al*. [13].

Mass spectra were collected in the reflection mode in a matrix-assisted laser desorption/ionization reflection time-of-flight MALDI-TOF mass spectrometer BIFLEX (Bruker-Franzen, Bremen, Germany). Analysis based on peptide mass fingerprinting at ProFound searching was performed.

### Tissue processing and peroxidase immunohistochemistry

Tissue processing and peroxidase immunohistochemistry was performed as described previously [9], with some modifications. Briefly, several 10 × 10 × 5 mm blocks of tissue were collected from different central cotyledons into cryomold biopsy vinyl specimen molds (Tissue Tek Cryomold Biopsy, Miles Inc. Division, Elkhart, IN) which were frozen by floating on liquid nitrogen and stored at -80°C until use. Frozen tissues were sliced into 7 μm serial sections and placed on a slide. The slides were dried for 16 hours in a hood, fixed for 10 minutes in acetone at room temperature, and stored at -20°C until the day of immunostaining. Before immunohistochemistry, the slides were transferred at -20°C into a box with drierite anhydrous calcium sulphate (W.A. Hammond Drierite Co, Ltd, Xenia, OH) to prevent water condensation during the equilibration at room temperature, and then incubated overnight (at 4°C) with non-diluted primary antibody TRO-tf-01. Control staining consisted of the replacement of the primary antibody with PBS, pH 7.22. The slides were then washed three times in PBS and incubated 20 minutes with the peroxidase conjugate swine anti-mouse IgG (SWAM/Px; Sevapharma, Prague, Czech Republic) diluted 1:50, washed again three times, and visualized with 3,3'-diaminobenzidine tetrahydrochloride followed by a slight hematoxylin counterstain [9].

Evaluation was performed under a Leitz DM RB (Leica Inc., Wetzlar, Germany) microscope equipped with differential interference contrast and a DEI-470 CCD Video Camera System (Optronics Engineering, Goleta, CA) with detail enhancement. The images were captured by a CG-7 color frame grabber (Scion Corporation, Frederick, MD) supported by Scion Image public software developed at the National Institutes of Health (Wayne Rasband, NIH, Bethesda, MD).

For the purpose of density measurement, the slides were immunostained for TF without hematoxylin counterstain. Five density measurements (area 100 square pixels) were performed for each placental sample. The resulting crude optical density (O.D.; scale 0–255) was subtracted from the mean of the O.D. background. Such "net O.D." values were used for statistical analysis.

### Statistical analysis

The net O.D. values were subjected to one-way ANOVA with Tukey-Kramer multiple comparisons post-test using the GraphPad InStat version 3.00 for Windows (GraphPad Software, San Diego, California, USA). Statistical significance was set at *p *< 0.05.

## Results

In a further study, we focused on the monoclonal antibody TRO-tf-01, which was found to specifically interact not only with JAR cells, but also with placenta lysates that exhibited differences in staining with respect to their origin. This antibody could also be used in immunohistochemical experiments on placenta tissue sections. Available clinical data of the pregnancies are shown in Table 1.

**Table 1:** Clinical data of the pregnancies.

	Age	W	Sex	mpl	m	BP	placenta type
1	24	37,6	F	620	3310	136/87	N
2	30	40,5	M	1000	5115	90/53	N
3	20	34,0	F	817	2220	112/65	N
4	22	36,4	M	526	2615	111/77	DrA
5	30	39,5	F	610	2955	199/85	DrA
6	32	36,6	M	645	2810	133/82	DrA
7	34	38,1	M	815	3800	117/59	GD
8	34	36,3	F	660	2805	115/71	GD
9	27	37,3	F	880	3415	109/74	GD
10	22	37,2	M	650	3665	141/75	PIH
11	32	39,3	F	600	3245	140/41	PIH
12	39	37,2	F	593	3180	140/78	PIH

NP, normal pregnancies; GD, gestational diabetes during pregnancy; PIH, pregnancy-induced hypertension during pregnancy; DrA, drug-abusing mothers during pregnancy.

w – week of gestation, M – male, F – female, mpl – weight of placenta, m – weight of newborn, BP – blood pressure

### Determination of antigen corresponding to MoAb TRO-tf-01

**Figure 1:**
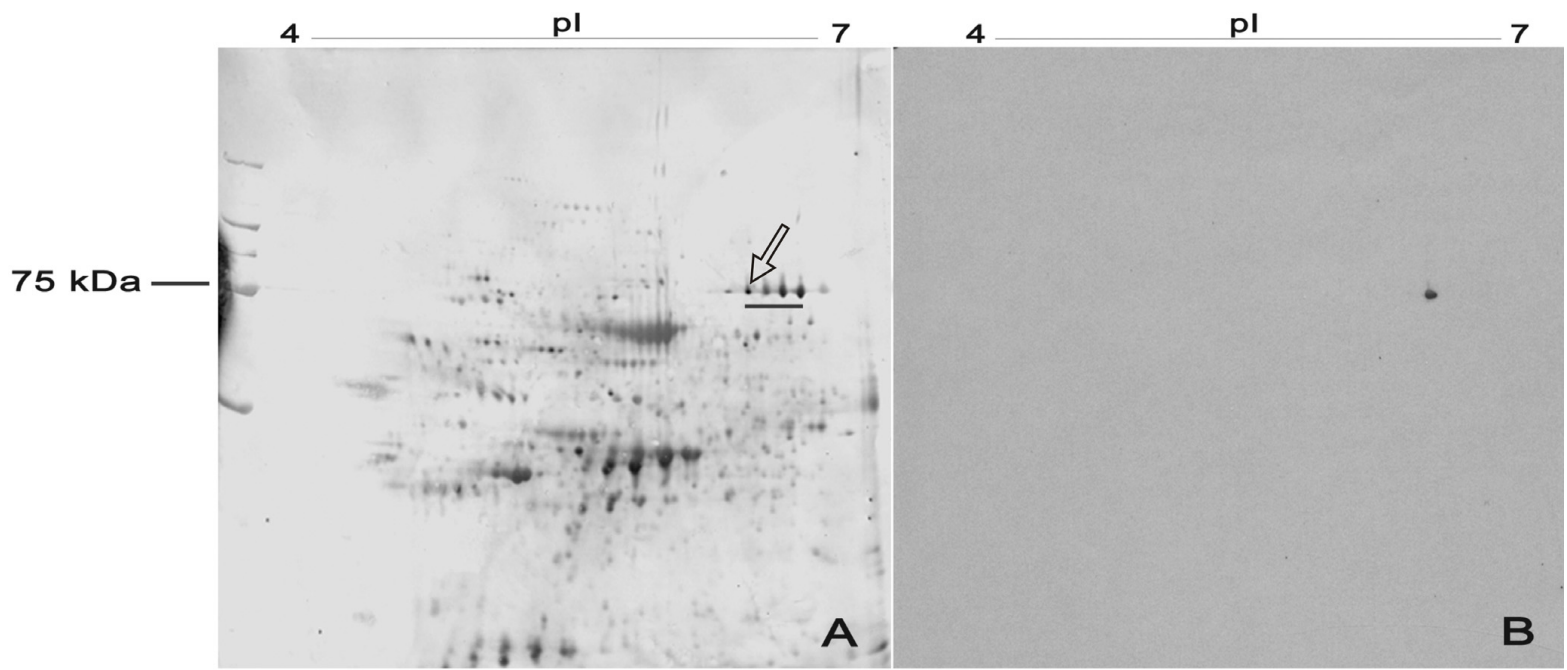
**Placenta lysate stained with CBB after 2D-SDS PAGE (A) and immunodetection with TRO-tf-01 antibody (B)**. A 13 cm Immobiline DryStrip of pH range 4–7 was used for the separation of the total protein lysate according to pI, in the second dimension, 10% polyacrylamide slab gel (SDS) was used. After immunoblotting with MoAb TRO-tf-01, only one isoform (arrow) of transferrins (underlined) was labeled (B). Detected antigen was identified by MALDI-TOF analysis as human transferrin (TF, sequence coverage of 31%, Z-score of 2.31). Molecular weight is shown on the left.

In 2-D electrophoresis, we used placenta lysates for antigen characterization (Figure 1). The positive ion mass spectra were measured in the reflection mode for the analysed proteins. The received values of m/z were used for protein identification after removing the matrix peaks and trypsin autoproteolytic peptides. The protein labeled by TRO-tf-01 was further defined by mass fingerprinting analysis. The obtained MALDI-MS spectra were interpreted by the ProFound database searching program [14]. The obtained peptides were matched with human transferrin peptide fragments (NP_001054.1, PRO2086, transferrin, Homo sapiens) with the sequence coverage of 31% and Z-score of 2.31. ProFound calculated the probability that a candidate in the database search is the protein being analyzed. As an indicator of the quality of the search result, a Z-score is estimated. A Z-score of 2.31 corresponds to the 99th percentile of the search in a random match population. Thus, mass spectrometric analysis showed that MoAb TRO-tf-01 is specific to one isoform of transferrin (TF), a glycoprotein of estimated molecular weight of 79.3 kDa and pI 6.9. Although MALDI-TOF analysis revealed more isoforms of transferrin that probably represent various posttranslational modifications of one protein (Figure 1A), only one isoform was detected with the TRO-tf-01 antibody (Figure 1B).

### Specificity of MoAb TRO-tf-01, immunobiochemistry

The monoclonal antibody TRO-tf-01 selected for the present study is of the IgG1 isotype, containing kappa light chains as determined by a mouse monoclonal antibody isotyping kit (IsoStrip, Roche, Indianapolis, USA).

**Figure 2:**
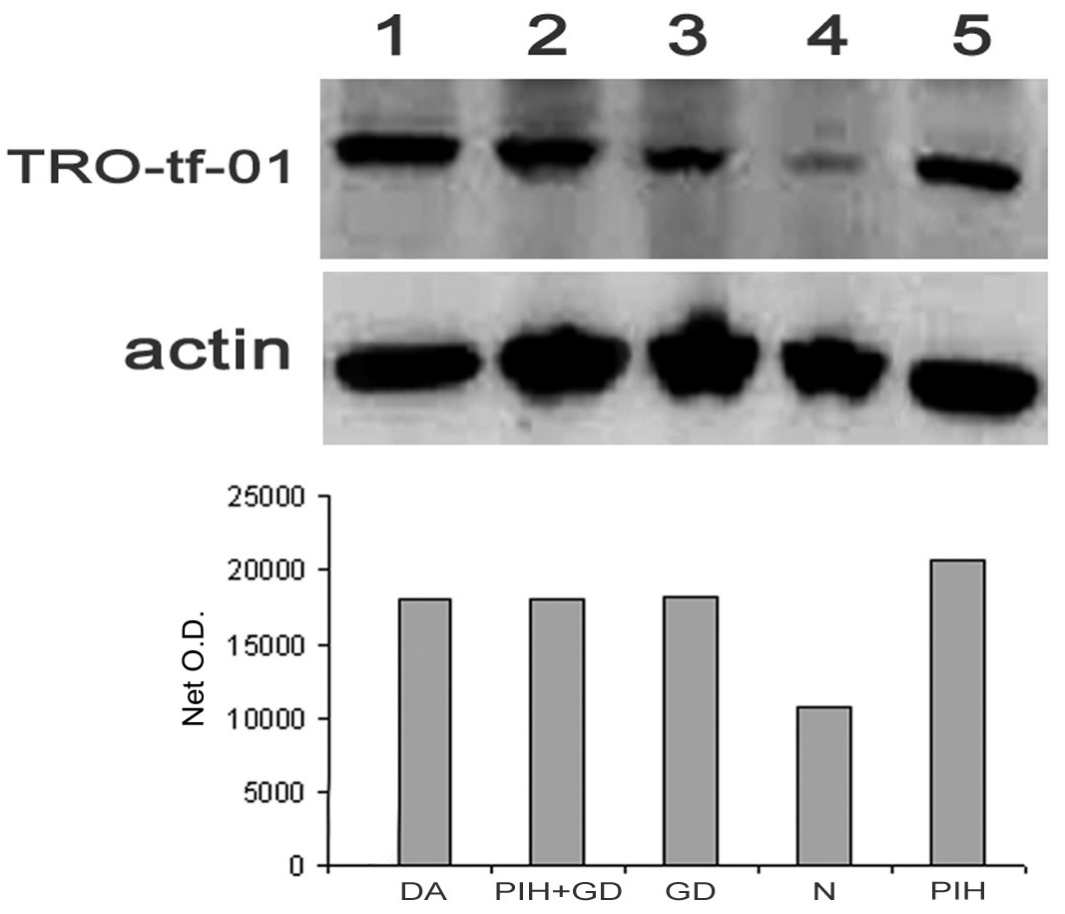
**Western blot analysis of chorionic villi of the placentae from normal and selected abnormal pregnancies with antibody TRO-tf-01**. Actin is used as a control for protein loading; lysates (NP-40) were loaded on 10% acrylamide gel (SDS, reducing conditions). Quantitative evaluation of TRO-tf-01 expression (O.D.) in the placentae is placed at the bottom. Placentae of mothers with 1 – drug abuse during pregnancy (DrA), 2 – pregnancy-induced hypertension together with gestational diabetes (PIH+GD), 3 – gestational diabetes (GD), 4 – normal pregnancy, 5 – pregnancy-induced hypertension (PIH).

Positive reactions of antibody TRO-tf-01 were gained in ELISA assay and Western blot analysis using NP-40 and SDS extracts of JAR cells and placental tissues (Figure 2), where MoAb labeled a protein of molecular weight ~80 kDa (Figure 1, 2).

According to the observed characteristics, the placenta samples were subdivided into two groups – normal and abnormal pregnancy (selected abnormalities – gestational diabetes, pregnancy induced hypertension, drug abusers). In biochemical experiments, expression differences of transferrin among placental samples were observed. In general, we noticed stronger reactions with TRO-tf-01 in placental samples originating from abnormal pregnancies (Figure 2, lane 1, 2, 3, 5) as opposed to the normal ones (Figure 2, lane 4).

Quantitative evaluation of TRO-tf-01 has shown a marked difference between net O.D. of the expression in the placentae of normal pregnancies and the placentae of mothers with gestational diabetes, pregnancy-induced hypertension, and drug abusers (Figure 2, bottom).

### Immunohistochemistry

**Figure 3:**
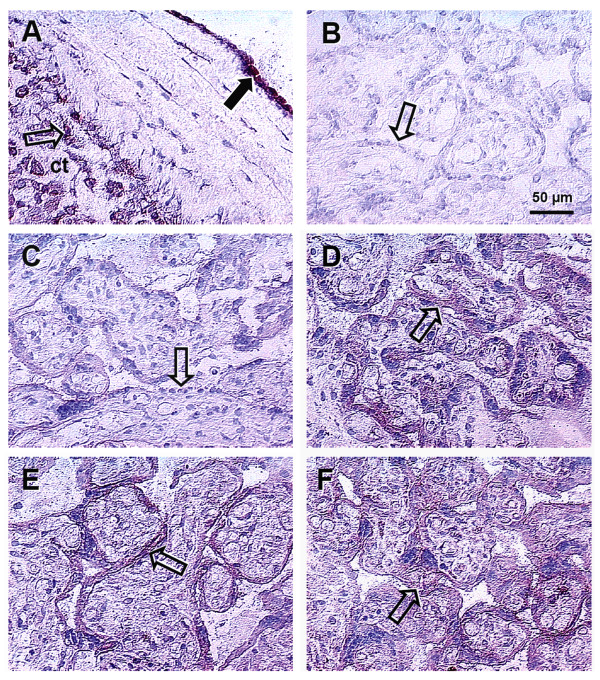
**Immunoreactivity of TRO-tf-01 in chorionic villi of placentae from normal and selected abnormal pregnancies**. A) Fetal membranes show very strong expression of transferrin by amniotic epithelium (full arrowhead) and on extravillous cytotrophoblast cells (arrowhead). B) Control experiment without primary antibody shows no transferrin staining in the syncytium of normal term placenta. C) Very low expression of transferrin in the syncytium of normal term placenta. D) Placenta of mother with gestational diabetes shows strong villous expression of transferrin. E) Placenta of mother with pregnancy-induced hypertension shows strong expression of transferrin in villous syncytium. F) Placenta of drug-abuse mother during pregnancy; the transferrin expression is very strong. Hematoxylin counterstained the nuclei.

Consistent strong expression of TF was found in all evaluated placentae in extravillous cytotrophoblast in the placental basal plate, fetal membranes (arrowhead, Figure 3A), and in amniotic epithelium (black arrowhead, Figure 3A).

However, differences in TF expression were detected between the placentae from normal and abnormal pregnancies in villous syncytiotrophoblast, which is in direct contact with maternal blood. In placentae from normal pregnancies, the expression of TF in syncytium was low (Figure 3C). Very strong expression was observed in syncytium of the placentae of mothers with gestational diabetes (Figure 3D), pregnancy-induced hypertension (Figure 3E), and drug abusers (Figure 3F).

**Figure 4:**
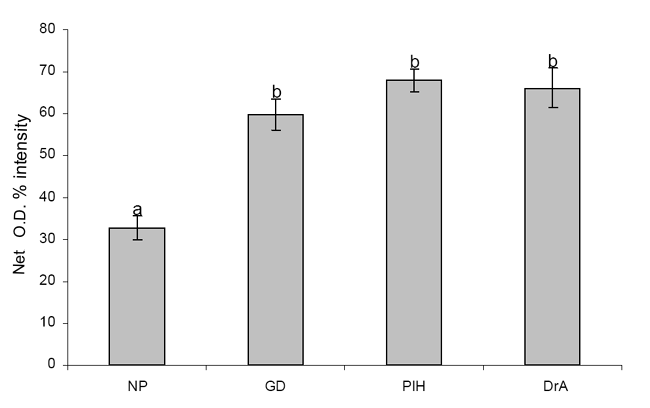
**Quantitative evaluation of TRO-tf-01 immunoreactivity in placental samples**. Each column represents mean (± SE) from fifteen net optical densities (Net O.D.) measured in three different placentae. NP, normal pregnancies; GD, gestational diabetes during pregnancy; PIH, pregnancy-induced hypertension during pregnancy; DrA, drug-abusing mothers during pregnancy. Statistical analysis has shown highly significant differences (p < 0.001) between normal (a) and each of the selected abnormal pregnancy types (b).

Quantitative evaluation of TRO-tf-01 immunoreactivity is presented in Figure 4. Statistical analysis has shown highly significant differences (p < 0.001) between the net O.D. of placentae from normal pregnancies and the placentae of mothers with gestational diabetes, pregnancy-induced hypertension, and drug abusers.

## Discussion

During implantation, the embryo shows an invasive phenotype and further, it eludes the immune system [15]. Thus, critical factors of maternal-fetal interaction seem to be of an immunological character [16]. Some of the immunological responses are of general importance; however, the majority of them are specific for maternal-fetal interactions [17,18]. It is obvious that the study of implantation and placenta formation is complex as many different cells and molecules participate in these processes. We can suppose that proteins, which are normally found at the maternal-fetal interface, are also important for successful embryo implantation, and their altered expression may therefore be connected with placental abnormalities. One of these significant proteins may be transferrin (TF).

As placental cells produce TF, it is supposed to function in transport or regulation of iron passage across this barrier [6]. Its defficiency (dysbalance) can result in some disorders influencing fetus development. There are various isoforms of transferrins in the placenta tissue ensuring iron supplementation of the fetus. It may be suggested that the increase of TF variants is correlated to an increase of iron transport [19]. According to the databases it is evident that the family of TFs exibits a great sequence variability with many aminoacid substitutions in different TF isoforms leading to a change of epitope properties. Moreover, in accordance with our findings, post-translational modifications of the TF epitopes (e.g. glycosylation) that differ in various cells and tissues were described [19]. All these variants can also change the TFs' functional properties to make the system of iron exchange even more effective. One of such variants might be the isoform detected by our antibody TRO-tf-01. According to the observations in placenta lysate, our MoAb TRO-tf-01 interacts with only one of the TFs (Figure 1) carrying a particular epitope which is not detectable in all TF forms. However, the phenomenon underlying this difference remains unclear for the present and needs to be elucidated.

A newly prepared MoAb against one isoform of TF reacted with JAR cells and placenta samples. In Western blot analysis, we found stronger reactions of all samples originating from placentae of selected abnormalities, when compared to the normal ones. However, these results themselves do not describe the placental status in the sense of abnormality, as various particular regions of placental tissue vary in the levels of TF expression. Therefore, these differences do not necessarily correspond with the placental status.

Thus, we used sections of well-characterized placentae for histological evaluation of TF expression patterns. Low expression of TF in the syncytiotrophoblast of the placentae from normal term pregnancies indicates existing saturation of fetal demands. Increased TF expression in the placentae from pregnancies of selected abnormalities may indicate an increased need for the TF function to meet the fetal iron needs. Hence, the enhanced expression of TF in the villous syncytiotrophoblast in selected abnormal pregnancy placentae may indicate an activation of an adaptive placental mechanism to satisfy the altered fetal demands for iron. In summary, we speculate that a strong expression of TF in the syncytiotrophoblast of selected abnormal pregnancy placentae is one of the responses to developing or existing fetal stress.

Morris Buus and Boockfor [6] described TF expression by both differentiated and non-differentiated placental cells, which strengthens the possibility that placental TF may be central to the passage of iron from the mother to the fetus during development. Moreover, according to Verrijt *et al*. [4], TF synthesis is not an exclusive property of the differentiated syncytiotrophoblast cells, suggesting that the state of cell differentiation might be just a determinant factor of the TF expression level. However, we observed consistent cytotrophoblast expression of TF in all used placenta samples, whereas the syncytiotrophoblast TF expression varied among various samples. As cytotrophoblast represents a precursor of syncytiotrophoblast and differentiates into syncytium during placenta formation, its function is important during implantation and early placentation. Therefore, the presence of TF in the extravillous cytotrophoblast suggests its possible involvement in such events.

Interestingly, strong expression of TF was also found in amniotic epithelium [4]. However its importance here remains unclear. Although there are reports showing evidence of TF production in the placenta [4,6], it does not necessarily mean that the increase in TF expression in the placentae after an abnormal pregnancy course is due to the increased production of TF in the cell or that TF in the cell is highly active. Another possible explanation is that the metabolism of TF in the syncytiotrophoblasts might be prevented. The explication can be connected with the presence of transferrin receptor (TfR, a transmembrane protein mediating the cellular iron uptake by binding and internalization of diferric transferrin) and its function that can influence the amount of TF in the placenta [20,21]. Several iron transporters and regulators were characterized recently. Interestingly, these iron transporters localized in placental trophoblast cells, mainly in recycling endosomes, were found to interact. It was also suggested that the level of intracellular iron may regulate both TfR expression and TfR trafficking/transcytosis in polarized cells [22]. In cultured cytotrophoblasts, TfR levels increase in cells cultured in iron-poor medium, indicating that iron has an effect on the TfR synthesis/breakdown ratio. These cultured trophoblasts regulate iron uptake by variation of the number of surface TfRs via changes in total TfRs and their redistribution in the membrane [23]. Also, *in vivo *the placenta minimizes the effect of the deficiency by up-regulating the proteins involved in Fe transfer. For example, TfR levels increase inversely to maternal Fe levels [24], which was described to occur in diabetic pregnancies that are complicated by low fetal iron stores. There the expression of TfR is increased, suggesting the regulation of placental iron transport by fetoplacental iron status [25].

Iron deficiency during pregnancy is common and has serious effects such as fetal growth retardation and cardiovascular problems in the adult offspring. Supplementation with iron is generally recommended during pregnancy to meet the iron needs of both mother and fetus. However, iron supplements and increased iron stores have recently been linked to maternal complications (e. g., gestational diabetes) and increased oxidative stress during pregnancy [7]. Consequently, while iron supplementation may improve pregnancy outcome when the mother is iron-deficient, it is also possible that prophylactic supplementation may increase the risk when the mother does not have iron deficiency or iron deficiency anemia [7].

The relation between drug abuse and iron metabolism is known. For instance, an increased level of iron also connected with pathological events such as alcoholic liver disease was described [26]. Even mild to moderate alcohol consumption has been shown to increase the prevalence of iron overload. Both iron and alcohol individually cause oxidative stress and lipid peroxidation, which culminates in liver injury. Despite these observations, the underlying mechanisms of iron accumulation remain unclear [26].

It is evident that a certain level of iron is important for the metabolism and proper cell proliferation and its dysbalance may be connected with various abnormal events in the organism. The importance of TF in trophoblast differentiation is well known; however, our present results indicate the possible involvement of a higher TF level, accompanying selected abnormal pregnancies, at the maternal-fetal interface.

## Competing interests

The authors declare that they have no competing interests.

## Authors' contributions

AK carried out the biochemical studies, participated in the antibody preparation and characterization, and drafted the manuscript. MS carried out the immunohistochemistry and performed the statistical analysis, AB participated in the immunohistochemistry and helped to draft the manuscript. JM and ZUG participated in the design of the study. JP conceived the study, participated in its design and coordination, and helped to draft the manuscript. All authors read and approved the final manuscript.
